# Supplementary material for: End Sequence Analysis Toolkit (ESAT) expands the extractable information from single-cell RNA-seq data
Source: Genome Res. 2016 Oct;26(10):1397–410. doi: 10.1101/gr.207902.116 (PMC5052061; doi:10.1101/gr.207902.116)
Supplement: Supplemental Material [file supp_gr.207902.116_Supp_Materials.tar.gz › ESAT-esat_v0.1/doc/html/classbroad_1_1pda_1_1gene_1_1_gene_tools.html]

ESAT: broad.pda.gene.GeneTools Class Reference


ESAT


- Main Page
- Related Pages
- Packages
- Classes
- Files

- Class List
- Class Index
- Class Hierarchy
- Class Members

All Classes Namespaces Files Functions Variables Enumerator Pages

- broad
- pda
- gene
- GeneTools

Static Public Member Functions |
List of all members

broad.pda.gene.GeneTools Class Reference

Collaboration diagram for broad.pda.gene.GeneTools:

[legend]

|  |  |
| --- | --- |
| Static Public Member Functions | |
| static void | main (String[] args) throws Exception |
|  | |
| static Map< String,   IntervalTree< Gene > > | merge (Map< String, Collection< Gene >> originalMap) |
|  | |
| static IntervalTree< Gene > | merge (Collection< Gene > original) |
|  | |
| static String | orientationForGene (Gene g, Sequence cSeq) |
|  | |
| static String | orientationFromSpliceSites (Annotation introns, Sequence seq, boolean onlyCanonical) |
|  | |
| static String | orientationFromSpliceSites (Annotation introns, Sequence chrSeq) |
|  | |
| static Map< String,   IntervalTree< Gene > > | overlap (Map< String, IntervalTree< Gene >> set1, Map< String, IntervalTree< Gene >>set2, Boolean byExon) |
|  | |
| static Map< String,   IntervalTree< Gene > > | mergeSets (Map< String, IntervalTree< Gene >> set1, Map< String, IntervalTree< Gene >> set2) |
|  | |
| static Gene | selectHighScoringIsoform (Gene transcript, BEDFileParser set) |
|  | |
| static BEDFileParser | mergeByGtfGeneId (String inFile, String outFile, String outFormat, String src, boolean writeMode) throws Exception |
|  | |
| static BEDFileParser | intersectGeneSets (Collection< Gene > set1, Collection< Gene > set2, boolean takeComplement) |
|  | |

## Member Function Documentation

|  |  |  |  |
| --- | --- | --- | --- |
| static BEDFileParser broad.pda.gene.GeneTools.intersectGeneSets | ( | Collection< Gene > | *set1*, |
|  |  | Collection< Gene > | *set2*, |
|  |  | boolean | *takeComplement* |
|  | ) |  |  |

static

|  |  |  |  |  |  |
| --- | --- | --- | --- | --- | --- |
| static void broad.pda.gene.GeneTools.main | ( | String[] | *args* | ) | throws Exception |

static

Here is the call graph for this function:

|  |  |  |  |  |  |
| --- | --- | --- | --- | --- | --- |
| static Map<String, IntervalTree<Gene> > broad.pda.gene.GeneTools.merge | ( | Map< String, Collection< Gene >> | *originalMap* | ) |  |

static

Here is the caller graph for this function:

|  |  |  |  |  |  |
| --- | --- | --- | --- | --- | --- |
| static IntervalTree<Gene> broad.pda.gene.GeneTools.merge | ( | Collection< Gene > | *original* | ) |  |

static

|  |  |  |  |
| --- | --- | --- | --- |
| static BEDFileParser broad.pda.gene.GeneTools.mergeByGtfGeneId | ( | String | *inFile*, |
|  |  | String | *outFile*, |
|  |  | String | *outFormat*, |
|  |  | String | *src*, |
|  |  | boolean | *writeMode* |
|  | ) |  | throws Exception |

static

Here is the caller graph for this function:

|  |  |  |  |
| --- | --- | --- | --- |
| static Map<String, IntervalTree<Gene> > broad.pda.gene.GeneTools.mergeSets | ( | Map< String, IntervalTree< Gene >> | *set1*, |
|  |  | Map< String, IntervalTree< Gene >> | *set2* |
|  | ) |  |  |

static

|  |  |  |  |
| --- | --- | --- | --- |
| static String broad.pda.gene.GeneTools.orientationForGene | ( | Gene | *g*, |
|  |  | Sequence | *cSeq* |
|  | ) |  |  |

static

Here is the call graph for this function:

|  |  |  |  |
| --- | --- | --- | --- |
| static String broad.pda.gene.GeneTools.orientationFromSpliceSites | ( | Annotation | *introns*, |
|  |  | Sequence | *seq*, |
|  |  | boolean | *onlyCanonical* |
|  | ) |  |  |

static

Here is the caller graph for this function:

|  |  |  |  |
| --- | --- | --- | --- |
| static String broad.pda.gene.GeneTools.orientationFromSpliceSites | ( | Annotation | *introns*, |
|  |  | Sequence | *chrSeq* |
|  | ) |  |  |

static

Here is the call graph for this function:

|  |  |  |  |
| --- | --- | --- | --- |
| static Map<String, IntervalTree<Gene> > broad.pda.gene.GeneTools.overlap | ( | Map< String, IntervalTree< Gene >> | *set1*, |
|  |  | Map< String, IntervalTree< Gene >> | *set2*, |
|  |  | Boolean | *byExon* |
|  | ) |  |  |

static

Here is the caller graph for this function:

|  |  |  |  |
| --- | --- | --- | --- |
| static Gene broad.pda.gene.GeneTools.selectHighScoringIsoform | ( | Gene | *transcript*, |
|  |  | BEDFileParser | *set* |
|  | ) |  |  |

static

---

The documentation for this class was generated from the following file:

- java/broad/pda/gene/GeneTools.java


---

Generated on Fri Aug 15 2014 15:10:59 for ESAT by  

 1.8.7
